# FGF8 coordinates tissue elongation and cell epithelialization during early kidney tubulogenesis

**DOI:** 10.1242/dev.122408

**Published:** 2015-07-01

**Authors:** Yuji Atsuta, Yoshiko Takahashi

**Affiliations:** 1 Department of Zoology, Graduate School of Science, Kyoto University, Kitashirakawa, Sakyo-ku, Kyoto 606-8502, Japan; 2 Core Research for Evolutional Science and Technology (CREST), Japan Science and Technology Agency (JST), Chiyoda-ku, Tokyo 102-0076, Japan

**Keywords:** Wolffian duct/nephric duct, Chemoattraction, Live imaging, Body axis

## Abstract

When a tubular structure forms during early embryogenesis, tubular elongation and lumen formation (epithelialization) proceed simultaneously in a spatiotemporally coordinated manner. We here demonstrate, using the Wolffian duct (WD) of early chicken embryos, that this coordination is regulated by the expression of FGF8, which shifts posteriorly during body axis elongation. FGF8 acts as a chemoattractant on the leader cells of the elongating WD and prevents them from epithelialization, whereas static (‘rear’) cells that receive progressively less FGF8 undergo epithelialization to form a lumen. Thus, FGF8 acts as a binary switch that distinguishes tubular elongation from lumen formation. The posteriorly shifting FGF8 is also known to regulate somite segmentation, suggesting that multiple types of tissue morphogenesis are coordinately regulated by macroscopic changes in body growth.

## Abstract (precis)

**Highlighted article:** Body axis elongation is regulated by posterior FGF8 signals . In chicken, nephric duct extension also requires this FGF8 signal, while low FGF8 anteriorly triggers duct lumen formation.

## INTRODUCTION

In many organs, tubular epithelia perform key physiological functions. For example, they convey food in the gut, exocrine factors from the pancreas and wastes from the kidney. For these functions, tubular integrity is important because its failure would cause pathological defects (Andrew and Ewald, 2010). To understand how such tubular integrity is established, it is necessary to delineate the mechanisms underlying epithelial tubule formation.

Tubule formation involves two major, coordinated steps: tubular elongation, a process that controls the length of the tubule; and cell epithelialization, a process that forms an internal lumen. These two events must take place in a spatiotemporally coordinated manner. However, how such coordination is achieved at the cellular and molecular levels has remained largely unexplored. This lack of knowledge is mainly due to the lack of experimental models that can simultaneously address the integrated questions regarding tubule elongation and epithelialization.

We have recently developed a novel technique of *in ovo* electroporation that enables gene manipulation of the forming Wolffian duct (WD; also called the nephric duct) in chicken embryos (Atsuta et al., 2013). The WD emerges in the anterior intermediate mesoderm (IMM) of the pronephric region, and subsequently extends caudally as a straight cord along a stereotypic path in between the presomitic mesoderm (PSM) and lateral plate (Obara-Ishihara et al., 1999; Saxén and Sariola, 1987). During WD elongation, the mesenchymal cord progressively hollows to form a single-layered epithelial tube through the process of mesenchymal-epithelial transition (MET). Importantly, cells located at the leader of the elongating WD (‘leader’ cells) are mesenchymal in shape and highly motile, as previously reported in chickens (Atsuta et al., 2013) and mice (Chia et al., 2011; Soofi et al., 2012), whereas ‘rear’ cells are epithelial and less motile (static).

Here, we studied how the mesenchymal and epithelial states are coordinately regulated in both time and space during WD elongation. We asked three questions: (1) what regulates the behavior of leader cells; (2) what determines the relative locations of the leader and static rear cells; and (3) what triggers epithelialization/lumenization? We found that FGF8, which is produced in a caudal region of the embryo (Dubrulle and Pourquie, 2004), plays crucial roles in these processes. FGF8 not only maintains the mesenchymal state of the leader cells, but also acts as a direct chemoattractant for their path finding. Since the FGF8-positive domain shifts caudally as the tail region elongates, the anteriorly positioned WD cells (i.e. rear cells) receive progressively less FGF8 signal, leading to their epithelialization and concomitant lumenization. Thus, tubule formation is harmonized with the growth rate of the embryo via FGF signals: mesenchymal and epithelial cells coordinately participate in elongation and lumenization, allowing tubule formation at the same rate as body axis elongation. Coordinated morphogenesis between the body axis elongation, WD elongation and somite segmentation is also discussed. Our results are in part consistent with those reported recently by Attia et al. (2015), who also showed the importance of FGF signals for WD elongation.

## RESULTS

### Tissue elongation is coordinated with cell epithelialization during WD formation

**Fig. 1.**
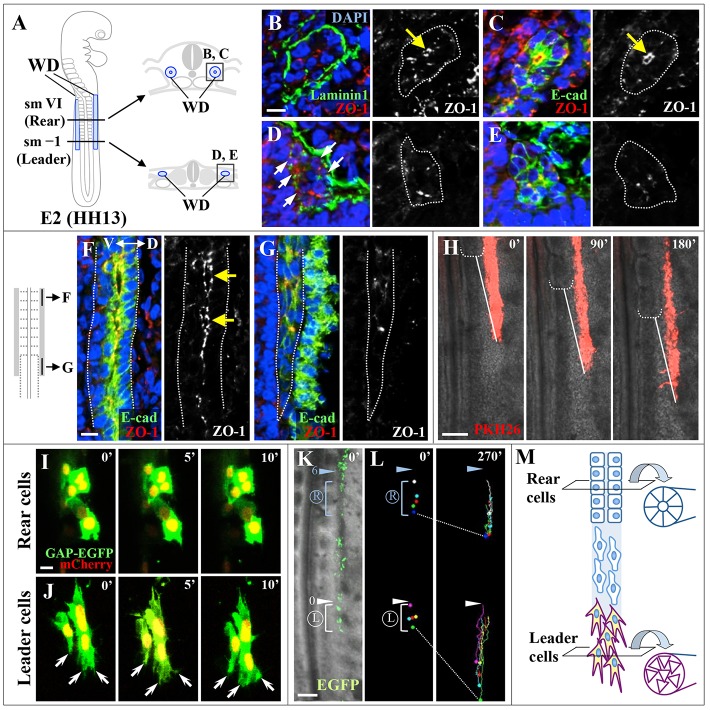
**Cell morphology of leader and rear cells in the forming WD.** (A) Drawings of whole-mount and transverse sections (in B-E) of an E2/HH13 chicken embryo. Cells located in the rear part [somite level (sm) V to VII] and the leader part (sm –2 to I) of the WD were termed rear cells and leader cells, respectively. (B-E) Transverse sections were stained with antibodies for laminin 1 and ZO-1 (B,D) or E-cadherin and ZO-1 (C,E). Yellow arrows (B,C) indicate apically localized ZO-1 in rear cells. White arrows (D) indicate sites where signals for laminin 1 are absent. Nuclei were stained with DAPI. (F,G) Sagittal sections of rear cells (F) and leader cells (G) were immunostained for E-cadherin and ZO-1. Yellow arrows (F) indicate lumens with apical ZO-1 signals. The E-cadherin-positive cell population seen dorsal to the WD is surface ectoderm. (H) Selected frames of an *in vivo* time-lapse movie (supplementary material Movie 1) showing the elongation of PKH26-labeled WD (red). White dotted brackets denote a newly formed somitic boundary. White solid lines indicate the interval between the white bracket and a tip of elongating WD. Note that the white lines in each panel are constant in length. (I,J) Selected frames from *in vivo* time-lapse movies (supplementary material Movies 2 and 3) showing magnified rear cells (I) and leader cells (J). Lamellipodia and filopodia were observed on leader cells (white arrows). (K,L) Migratory tracks of rear cells and leader cells are bracketed by blue and white lines, respectively. The light blue and white arrowheads indicate the sixth and newly formed somitic boundaries, respectively. (M) Diagram illustrating differential cell morphology in the elongating WD of the E2/HH13 chicken embryo. Leader cells are mesenchymal in shape and highly motile, whereas rear cells are static and constitute an epithelial tubule. Time is in minutes. Scale bars: 20 μm in B,F,I; 100 μm in H,K.

It is known that the WD emerges from the anteriorly located pronephric region of HH10 chick embryos, spanning the sixth to twelfth somite levels (Hiruma and Nakamura, 2003). Subsequently, the WD extends posteriorly as a simple straight cord, and this elongation is in register with somitic segmentation: the leader of the extending WD is constantly located in the PSM (unsegmented) at the level of one to two presumptive somites posterior to the most recently formed somite [somite level (sm) –1 to –2] (Atsuta et al., 2013; Saxén and Sariola, 1987). We found in HH13 embryos that the cells at the leader of the WD were mesenchymal with no tubular structure, whereas those located anterior to sm V (the fifth somite anterior to the forming somite) were part of an epithelial tubule. In a transverse view, WD cells at sm V were enclosed by the basal marker laminin 1, a component of the extracellular matrix (ECM), and exhibited apicobasal polarity as revealed by the tight junction marker ZO-1 and E-cadherin (Fig. 1A-C; *n*=10). By contrast, in the leader cells at sm –1, signals for laminin 1 and ZO-1 were sparse, with no obvious apicobasal polarity of E-cadherin signals (Fig. 1D,E; *n*=10). These observations were also supported by observation of sagittally sectioned specimens (Fig. 1F,G; *n*=10). In this study, we defined leader cells as those located between sm –2 and sm I, and rear cells as those between sm V and sm VII. Although leader cells exhibit sparse and weak laminin 1 and ZO-1 signals, we describe the leader cells as ‘mesenchyme’, so that morphological differences between leader cells and rear cells (epithelial) can be clearly highlighted.

To examine how the leader and rear cells behave in embryos, we performed time-lapse live imaging confocal microscopy at high resolution. During WD elongation, which progresses in register with somite segmentation (Fig. 1H; supplementary material Movie 1; *n*=3 embryos), leader cells were elongated in shape and possessed numerous cellular processes that repeatedly extended and retracted in the direction of WD elongation (Fig. 1J; supplementary material Movie 3). By contrast, static cells were polygonal in shape with few cellular processes, as is characteristic of epithelial cells (Fig. 1I; supplementary material Movie 2). In addition, long-term (270 min) tracing of WD cells demonstrated that leader cells were highly motile compared with rear cells (Fig. 1K,L; *n*=3 embryos).

The spatial relationship between the mesenchymal (leader) and epithelial (rear) cells was maintained regardless of the number of somites (embryonic stages), at least until HH16 (data not shown). Thus, these two types of cells were coordinated during WD elongation. Since the mesenchymal cells at the leader and the epithelial cells in the rear would serve for tubule elongation and lumenization, respectively, we reasoned that mesenchymal-epithelial coordination between the leader and rear would be crucial for correct WD formation in terms of size and shape.

### Leader cells are attracted to their adjacent intermediate mesoderm

We explored the cellular and molecular mechanisms by which spatiotemporal coordination is achieved between the motile leader cells and epithelial static cells during WD elongation. To specifically address the differential regulation of these two cell types, we analyzed how the motility of leader cells is regulated in the context of interactions between adjacent tissues. Unlike the situation reported in axolotl embryos (Morris et al., 2003), we found that the surface ectoderm appears to be dispensable, since blockage of ectodermal signals by removing this tissue (supplementary material Fig. S1A-C; *n*=12/15) or by inserting a piece of aluminium foil underneath this tissue (supplementary material Fig. S1D-E′; *n*=7/9) did not affect elongation of the WD, at least until HH15.

**Fig. 2.**
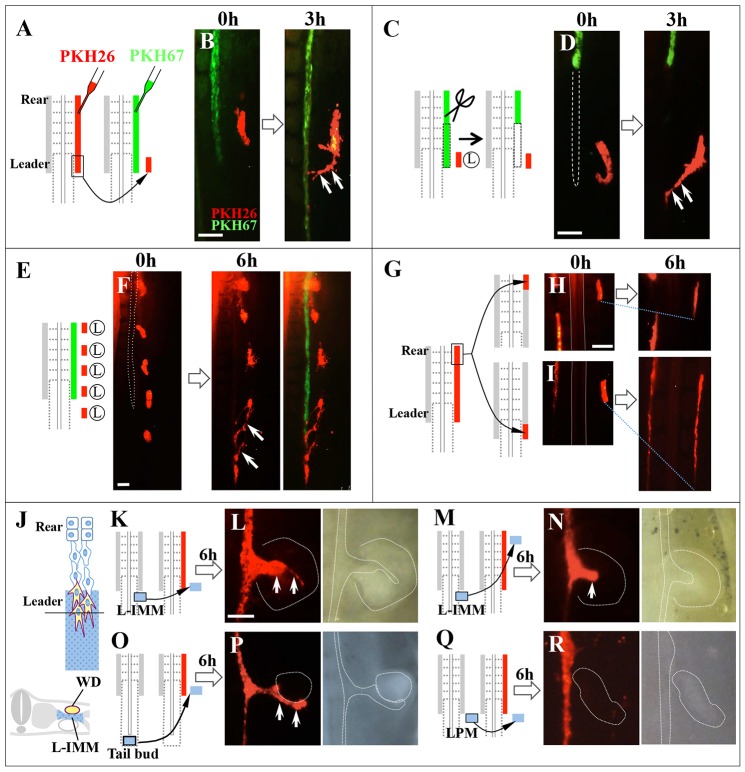
**WD cells are attracted to IMM that neighbors leader cells.** (A) PKH26-labeled leader cells (red) were transplanted into a region lateral to PKH67-labeled leader cells (green) of an E2/HH13 host embryo. (B) Donor cells (red) moved toward the host WD by 3 h after transplantation (arrows). (C) Leader cells (red) were grafted (similarly to B) into a host embryo from which leader cells had been removed. (D) The grafted leader cells moved mediocaudally (arrows), even if WD was not present. (E) Leader cells from five donor embryos (red) were transplanted into five different positions along the AP axis in the lateral plate mesoderm (LPM). (F) The posteriorly placed cells moved toward the leader area of host WD (arrows). (G-I) Rear cells of donor WD (red) were transplanted into the leader region (I) or rear region (H) of host embryos. (I) The transplant resumed marked elongation in the leader region. (J) The IMM adjacent to the leader cells was termed ‘L-intermediate mesoderm’ (L-IMM) (blue). (K,L) A piece of L-IMM was transplanted into LPM located medial to the leader cells. Arrows (L) indicate an ectopic branch from the host WD (red). (M,N) L-IMM also caused ectopic branch formation from the rear WD when relocated to this region (arrow). (O,P) A piece of tail bud was transplanted into LPM, resulting in the formation of an ectopic branch (arrows). (Q,R) LPM transplantation as a negative control. Scale bars: 100 μm.

To determine which tissue(s) regulates WD elongation, we ectopically placed a piece of leader WD into the lateral plate region (Fig. 2A). The grafted WD, which was labeled by PKH26, was attracted medially toward the IMM of host embryos (Fig. 2B; *n*=17/23). This attraction was also observed in a similar experiment in which a host WD had been ablated (Fig. 2C,D; *n*=7/9), suggesting that the IMM might be a primary source of attracting signals for the leader cells. To locate precisely the leader cell-attracting site in the IMM along the anteroposterior (AP) axis, leader cells dissected from embryos were PKH26 labeled and placed into five different positions along the AP axis in the lateral plate of the host embryo (Fig. 2E). We found that only the two posterior transplants moved to the IMM (Fig. 2F; *n*=4/4). Thus, during normal development the IMM near the leader cells, but not the IMM near the rear, provides cues for WD elongation. Of note, even the rear WD cells reacted to the leader-attracting signal: when relocated to the leader position of WD, the rear cells resumed migration (Fig. 2G,I; *n*=5), whereas this phenomenon was not observed when they were placed back in original position as a control (Fig. 2G,H; *n*=5). Thus, even quiescent epithelial WD (rear) cells can resume migration if they encounter attractive signals. Hereafter, we refer to the IMM region that surrounds the leader cells as ‘leader IMM’ (L-IMM) (Fig. 2J).

To further examine the leader cell-attracting activity exerted by L-IMM, we grafted a piece of L-IMM into a region adjacent to the leader WD of a host embryo (Fig. 2K). This resulted in ectopic branching of the host WD, which migrated toward the graft (Fig. 2L; *n*=12/27). Furthermore, the grafted L-IMM also induced ectopic branching from quiescent epithelial tube from the rear WD (approximately sm VI) (Fig. 2M,N; *n*=6/12). Thus, the L-IMM exerts attracting activities to both leader and rear WD. Such activity was not seen when lateral plate mesoderm (LPM) was used as a control (Fig. 2Q,R; *n*=0/9).

Together, these observations suggest that WD elongation is regulated by interactions between the WD leader and its adjacent L-IMM. The L-IMM exerts activities that might induce or promote the motility and migration of leader cells, and such activities must be located specifically to the region surrounding the leader cells.

### FGF signaling is activated in the elongating WD

To identify the WD-attracting signaling molecule(s) elicited by L-IMM, we focused on fibroblast growth factor (FGF) signals as candidates for the following reasons. First, FGF8 is known to be expressed in PSM (Dubrulle et al., 2001; Dubrulle and Pourquie, 2004). Second, a tail bud, also known to express FGF8 (Dubrulle and Pourquie, 2004), was found to induce ectopic deviation of the WD when placed into the lateral plate (Fig. 2O,P; *n*=5/7). Third, FGF signals are known to be essential for tubular branching of trachea in *Drosophila* (Sutherland et al., 1996) and lung and mammary glands in mammals (Andrew and Ewald, 2010; Lu and Werb, 2008).

**Fig. 3.**
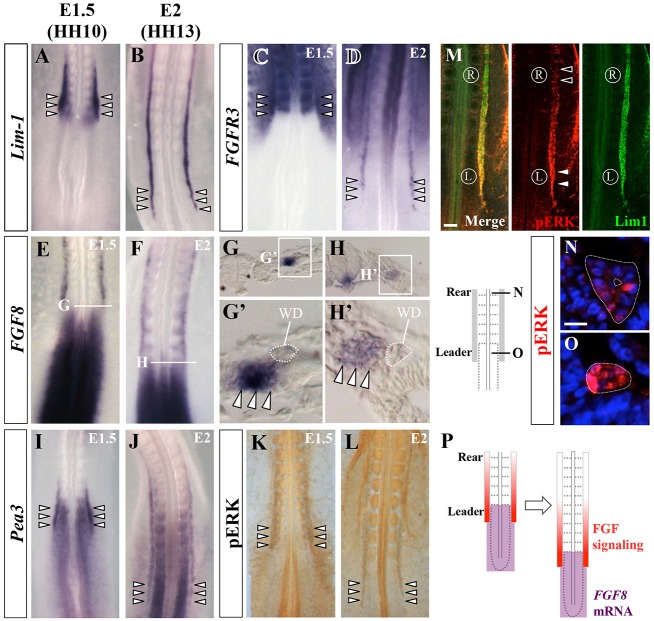
**Expression patterns of *FGF8*, *FGFR3* and *PEA3*, and ERK1/2 activity in WD.** (A-J) *In situ* hybridization to show expression of *LIM1* (A,B; a marker for WD), *FGFR3* (C,D), *FGF8* (E-H′) and *PEA3* (I,J) mRNA in E1.5/HH10 and E2/HH13 embryos. Arrowheads (A-D,I,J) indicate leader cells. The anterior border of the *FGF8-*expressing area, including L-IMM and PSM, was juxtaposed to the leader cells with a slight overlap (G′,H′, arrowheads). (K,L) Whole-mount immunostaining for diphosphorylated ERK1/2 (pERK) in E1.5/HH10 (K) and E2/HH13 (L) embryos. Arrowheads indicate leader cells. (M) E2/HH13 embryo stained with antibodies for pERK (red) and LIM1 protein (green). Signals for pERK were more intense in leader cells (white arrowheads) than in rear cells (black arrowheads). (N,O) Transverse sections of E2/HH13 embryos immunostained for pERK showing leader cells (O) and rear cells (N). (P) Diagram illustrating the relative positions of FGF signal activation in WD (red) and caudal regression of the *FGF8*-expressing area (purple). Scale bars: 100 μm in M; 20 μm in N.

We found that mRNAs of FGF receptor 1 (*FGFR1*), *FGFR2* (supplementary material Fig. S2A-D) and *FGFR3* (Fig. 3C,D) were localized in the elongating WD. In addition, as previously reported (Dubrulle et al., 2001; Dubrulle and Pourquie, 2004), *FGF8* was markedly expressed in the posterior region of the body, and this expression shifted posteriorly as the body axis elongated. Importantly, this *FGF8*-positive area included not only PSM, but also IMM (Fig. 3E-H′). Thus, the posterior shift of the FGF8-positive zone was in register with WD elongation (Fig. 3P).

To determine whether FGF signaling was activated in the WD leader, we examined the expression patterns of *PEA3* and *SEF*, which are downstream factors of FGF signaling. We found that they were expressed in WD at HH10 (Fig. 3I; supplementary material Fig. S2E) (Lunn et al., 2007), and also at HH13 (Fig. 3J; supplementary material Fig. S2F). We also detected phosphorylation of ERK1/2 (pERK), a prominent marker of FGF signaling, in the elongating WD (Fig. 3K,L). Notably, pERK signals were more intense in the leader cells than in rear cells (Fig. 3M-O). These observations suggested that the leader cells, but not the rear cells, receive FGF ligands, and also raised the possibility that the posteriorly shifting FGF8 would direct WD elongation by activating the leader cells (Fig. 3P).

### FGF8 is sufficient to attract leader cells of the WD

**Fig. 4.**
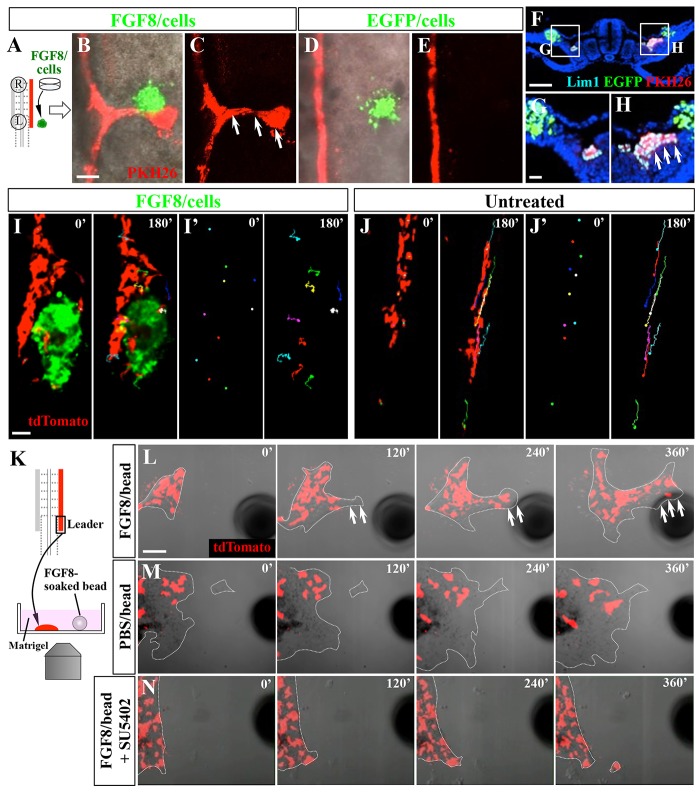
**FGF8 acts as a chemoattractant for WD cells.** (A-C) When an aggregate of FGF8-producing DF1 cells (green) was implanted lateral to the WD (red) of E2/HH13 embryos (A), ectopic branching of WD toward FGF8-producing cells (green) was observed (B,C, arrows). (D,E) EGFP-expressing cells (green) did not induce WD branching. (F-H) Transverse views of E2/HH13 embryos into which FGF8/cells (right, H) and EGF/cells (left, G) were implanted. WD was branched toward FGF8-producing cells (arrows in H) but not toward EGF/cells. (I,I′) Selected frames from an *in vivo* time-lapse movie showing the movement of tdTomato-electroporated WD cells (red), which were attracted to implanted FGF8-producing cells (supplementary material Movie 4). (J,J′) Cell tracking in an untreated embryo (supplementary material Movie 5). (K-N) *In vitro* chemoattraction assays. (K) Leader cells taken from tdTomato-electroporated WD were co-cultured either with an FGF8-soaked bead (L; supplementary material Movie 7), a PBS-soaked bead (M; supplementary material Movie 8), or an FGF8-soaked bead in the presence of 2 μM SU5402 (N; supplementary material Movie 9). The cells were attracted to the FGF8-soaked bead (L, arrows), whereas SU5402 abolished this attraction activity (N). Scale bars: 100 μm.

To determine whether FGF8 would act as a chemoattractant for WD cells, we implanted an aggregate of FGF8-producing DF1 cells (a chicken fibroblast cell line) into LPM. This induced ectopic deviation of the WD toward the implant (Fig. 4A-C; *n*=38/56). Similar manipulations using EGFP-transfected DF1 cells as a control did not affect WD elongation (Fig. 4D,E; supplementary material Fig. S3E). Ectopic deviation was also observed when FGF8-soaked beads were used in a similar way (supplementary material Fig. S3F). In these embryos, implants (cell aggregates or beads) were placed in between the somatopleural (lateral) mesoderm and surface ectoderm (Fig. 4F-H).

Since FGFs are known to promote cell proliferation, we asked if the ectopic deviation of WD that we observed was achieved by cell proliferation or cell migration. *In vivo* time-lapse analyses spanning 180 min revealed that a majority of WD cells migrated toward the implant of FGF8/DF1 without significant cell division (Fig. 4I,J; supplementary material Movies 4 and 5). Thus, the FGF8-induced deviation of the WD is achieved by chemoattractive activity with little contribution from cell proliferation.

We further tested whether the WD migration was mediated by a direct effect by FGF8. We conducted *in vitro* time-lapse imaging analyses in which leader cells were dissected from E2/HH13 embryos and co-cultured with FGF/DF1 cells (Fig. 4K). As shown in supplementary material Movie 6, WD cells migrated toward FGF/DF1 cells, which were also motile. Similarly, WD cells were attracted to an FGF8-soaked bead (Fig. 4L; supplementary material Movie 7). By contrast, there was no directional migration toward a control PBS-soaked bead, although the cells were motile (Fig. 4M; supplementary material Movie 8). Furthermore, the FGF8-induced attraction was inhibited in the presence of SU5402, an inhibitor of FGF signaling (Fig. 4N; supplementary material Movie 9). Together, we concluded that FGF8 acts as a chemoattractant for WD leader cells.

We also examined the effects of FGF4 and FGF10, transcripts of which were found in the tail bud and PSM, respectively (supplementary material Fig. S3A-D) (Karabagli et al., 2002). A bead soaked with FGF4 protein and FGF10-producing DF1 cells were implanted in between the surface ectoderm and somatopleure (in a similar manner to Fig. 4A and supplementary material Fig. S3F). Whereas FGF4-soaked beads induced deviation of the WD leader cells, no effect was seen with FGF10-producing cells (supplementary material Fig. S3G,H). These observations raise the possibility that FGF8 and FGF4 may act redundantly, which would also account for the previously reported phenomenon that WD elongation is unaffected in mice lacking FGF8 (Perantoni et al., 2005).

Although glial cell line-derived neurotrophic factor (GDNF) has been shown to direct WD cells in axolotl (Drawbridge et al., 2000), we found no effects in chick of GDNF/cells implanted near the WD (supplementary material Fig. S3I). This is consistent with previous reports for mice showing that GDNF is not involved in early WD elongation (Chi et al., 2009; Chia et al., 2011).

### FGF signals in the elongating WD through a Ras/ERK cascade

**Fig. 5.**
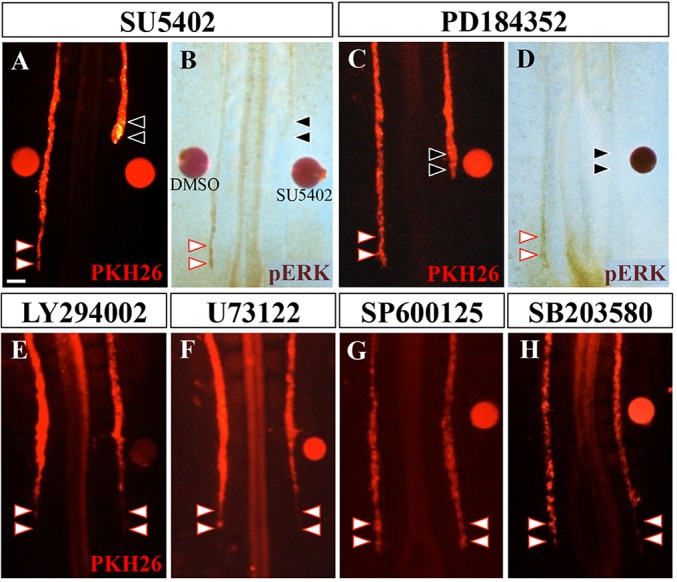
**FGF/ERK signaling was required for the WD elongation.** Beads soaked with either DMSO (A,B), SU5402 (FGFR inhibitor; A,B), PD184352 (MEK inhibitor; C,D), LY294002 (PI3K inhibitor; E), U73122 (PLCγ inhibitor; F), SP600125 (JNK inhibitor; G) or SB203580 (p38 inhibitor; H) were implanted into E2/HH13 embryos. WD elongation was disturbed by SU5402 or PD184352 beads (A,C, black arrowheads). At the posterior end of the disturbed WD, the pERK signal (brown) was markedly diminished (B,D, black arrowheads). Scale bars: 100 μm.

To determine whether FGF signals are essential for WD elongation, an SU5402-soaked bead was implanted into the lateral region. This treatment markedly disturbed WD elongation, whereas the control bead soaked with DMSO had no effect (Fig. 5A,B; *n*=22/30 for SU5402, *n*=0/30 for control). The expression of *LIM1*, an early marker for WD differentiation (Kobayashi et al., 2004; Tsang et al., 2000), was unaffected by SU5402 (supplementary material Fig. S4A,B; *n*=7). In addition, SU5402 exerted little effect on cell survival, as revealed by TUNEL assay (supplementary material Fig. S4C-F). Together, the disturbed elongation of the WD upon SU5402 treatment appears to be attributable to inhibited migration of the leader cells and not to any delayed differentiation of WD cells or enhanced cell death.

We further explored intracellular signaling of FGF in WD leader cells. Notably, in the SU5402-treated cells, pERK staining for was markedly diminished (Fig. 5B). To examine whether the FGF/ERK pathway is crucial for WD elongation, PD184352, an inhibitor of MEK that blocks ERK phosphorylation, was tested in a similar manner to the SU5402 bead treatment. PD184352 profoundly impeded WD elongation (Fig. 5C,D; *n*=6/11). We also tested four other drugs, namely LY294002 (PI3K inhibitor), U73122 (PLCγ inhibitor), SP600125 (JNK inhibitor) and SB203580 (p38 inhibitor), and none yielded appreciable effects (Fig. 5E-H; *n*=0/10 for each). Thus, we conclude that FGF signaling through the ERK pathway is crucial for controlling WD elongation.

### FGF-deprived WD cells undergo epithelialization to form a tubular structure

**Fig. 6.**
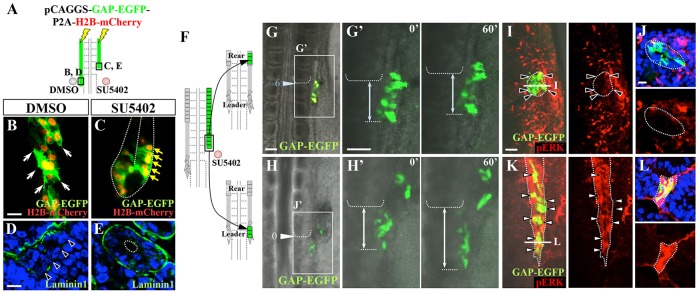
**FGF signaling regulates a binary decision between motile and epithelial cell states during WD elongation.** (A-E) FGF signaling affects cell morphology of the WD. (A-C) Leader cells were electroporated with pCAGGS-GAP-EGFP-P2A-H2B-mCherry, and treated with either DMSO- or SU5402-soaked beads. Leader cells treated with SU5402 were columnar epithelial (C, yellow arrows), whereas cells of DMSO-treated WD were mesenchymal (B, white arrows). (D,E) Transverse sections of SU5402-treated (D) or DMSO-treated (E) leader cells. SU5402-treated leader cells were enclosed by laminin 1, a marker of basement membrane. The light blue and white arrowheads indicate the sixth and newly formed somitic boundaries, respectively. (F-H′) Leader cells, which had been epithelialized after SU5402 treatment, were transplanted into the leader (H) or rear (G) regions of E2/HH13 embryos. Epithelial cells transplanted into the leader region resumed caudal migration (H′; supplementary material Movie 10), whereas those transplanted into the rear region remained static (G′; supplementary material Movie 11). (I-L) FGF signals after transplantation of SU5402-epithelialized leader cells (green) into normal embryos. Whereas cells that remained static in the rear transplantation exhibited no or little activation of pERK, cells transplanted into the leader region reactivated pERK (red) after 6 h (white arrowheads). Black arrowheads indicate that GAP-EGFP-electroporated cells, which were grafted into the rear region, are negative for pERK. Time is in minutes. Scale bars: 20 μm in B,D; 100 μm in G,G′,I.

We observed that SU5402-treated WD leader cells not only cease migration, but also undergo precocious epithelialization to form a tubular structure. An elongating WD, electroporated with GAP-EGFP (plasma membrane) and H2B-mCherry (nucleus), was treated with an SU5402-soaked bead (Fig. 6A-C). SU5402-treated cells were columnar in shape with their nuclei located along the perimeter of the tissue (Fig. 6C), a feature that is characteristic of epithelial tubules seen only in the rear position. This notion was further supported by observation of transverse views with laminin staining as a marker for basement membrane, which showed that SU5402-treated leader cells were apicobasally polarized (Fig. 6D,E). Thus, FGFs coordinate leader migration with tubular epithelialization during WD formation by acting as a molecular switch: in the presence of FGFs, WD cells are endowed with migratory and motile properties, whereas when deprived of FGFs they undergo tubular epithelialization.

### Epithelialized cells convert to migratory cells when placed in the leader region

As shown above, rear WD cells (tubular epithelium) can be converted to leader-like migratory cells when relocated to the leader region (Fig. 2G) or provided with a leader environment (Fig. 2M). To further examine the potential of tubular-to-migratory conversion, we exploited SU5402-treated leader cells, which underwent precocious epithelialization, since these cells are amenable to more detailed analyses than is possible with rear cells. We carried out time-lapse imaging of these cells (GAP-EGFP electroporated) when transplanted into either the leader or rear positions of elongating WD (Fig. 6F). When placed into the leader, SU5402-treated cells resumed migration with mesenchymal character (Fig. 6H,H′; supplementary material Movie 10). These cells also exhibited intense pERK signal (Fig. 6K,L). By contrast, when transplanted into the rear region, the cells retained their epithelial morphology with little motility (Fig. 6G,G′; supplementary material Movie 11), and exhibited little pERK signal (Fig. 6I,J).

Together, the FGF signal acts as a binary switch between migratory leader cells and quiescent epithelial tubular cells. Thus, the progressive posterior shift of FGF8 expression coordinates leader elongation with tubule formation during WD formation.

## DISCUSSION

**Fig. 7.**
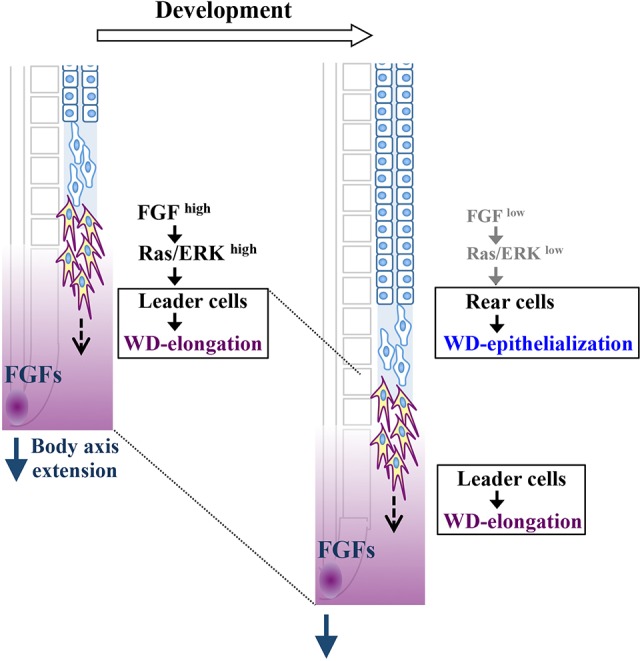
**Summary of the mechanisms underlying WD elongation as regulated by multiple types of cell and tissue coordination.** WD leader cells are attracted to nearby L-IMM, and this attraction is mediated by FGF activities initially produced from the tail bud of the body (Dubrulle and Pourquie, 2004). As the body axis extends, the FGF-expressing area (purple) shifts caudally, underlying the coordinated elongation of the WD and body axis. FGFs act as a binary switch in cellular morphogenesis within the elongating WD: high FGF activity confers high motility on leader cells, whereas rear cells, which are progressively released from the influence of the FGF-positive region, undergo epithelialization. Collectively, this scheme highlights how a macroscopic change in body axis elongation is coordinately translated into morphogenetic changes at the microscopic level in terms of the chemoattraction of leader cells and tubule formation of rear cells, both of which progress posteriorly with a regular interval between them.

By exploiting the simple structure of the WD and the amenability of tissue manipulations in chicken embryos, we have demonstrated that the WD is formed through a remarkably coordinated morphogenesis of tubule elongation and cell epithelialization. This coordination is mediated by a graded activation of FGF8 signals: posteriorly high signals chemoattract the leader cells, whereas anteriorly low signals enhance the tubular epithelialization of rear cells (Fig. 7). The posterior-to-anterior gradient of FGF8 is initiated by *FGF8* transcription in the tail bud, and, as the body axis elongates posteriorly, the *FGF8*-positive zone is likewise progressively displaced posteriorly (Dubrulle et al., 2001). Thus, body elongation coordinates WD formation by the tail-derived FGF signals, which are consequently translated into the morphological difference between the leader and rear cells, both of which progress posteriorly with a regular interval between them (Fig. 7). Since FGF8 expression in the tail region appears to be evolutionarily conserved in vertebrates, FGF8-mediated elongation of the WD/nephric duct is also likely to be conserved.

Another important finding of this study is that FGF8 acts as a binary switch between motile (leader) cells and epithelial (rear) cells (Fig. 7). High activity of FGF8 confers motility on the leader cells, which are responsible for tubular elongation. The rear cells, by contrast, when released from the FGF8-positive zone, undergo epithelialization and lumenization to form a tubular structure. Intriguingly, even the leader cells can be experimentally epithelialized by FGF deprivation (Fig. 6). How FGF deprivation leads to epithelialization awaits further investigation.

### FGF8 signaling in migrating WD cells

We have demonstrated that FGF8 acts as a chemoattractant and induces directional migration of WD cells both in embryos and in primary cultured WD cells *in vitro* (Fig. 4; supplementary material Movies 6 and 7). Notably, it seems that leader cells attracted to FGF8 are not fully determined, and other cells can become leader cells (supplementary material Movie 6; Y. A., unpublished). These phenomena are reminiscent of other leader/tip cells reported for blood vessel sprouting (Jakobsson et al., 2010) and neural crest migration (Wynn et al., 2013). Furthermore, epithelialized rear cells in the WD can be converted to motile leader cells when relocated into the leader environment without any substantial effects on cell proliferation, and this conversion is likely to be mediated by FGF8. Thus, the newly epithelialized tubular structure is naïve and susceptible to an FGF8-mediated EMT action. A few examples of the chemoattractive activities of FGFs have been shown previously, including cell migration along the primitive streak controlled by FGF4 (not by FGF8) (Yang et al., 2002) and neurite extension of motor neurons by FGF8 (Shirasaki et al., 2006).

It is likely that the FGF8 signaling is transmitted via FGFR3, transcripts of which are detected in the elongating WD. This might also account for the selective attraction of the leader cells to FGF8, as FGF8 binds to FGFR3 with higher specificity than to FGFR1 and FGFR2 (Zhang et al., 2006). Furthermore, it is likely that FGF4 acts redundantly with FGF8 during WD elongation, consistent with mouse genetic studies showing cooperative actions between FGF4 and FGF8 in limb and body axial outgrowth (Boulet and Capecchi, 2012; Boulet et al., 2004; Naiche et al., 2011; Sun et al., 2002). Intracellularly, the FGF8 signal during WD elongation is transmitted via the Ras/ERK pathway, whereas PLCγ and the PI3K pathways are dispensable (Fig. 5). Of note, sprouty 1 appears to be expressed in chicken WD (Geisha, http://geisha.arizona.edu/geisha/) (Antin et al., 2014). Since sprouty 1 is known to inhibit the PLCγ but not Ras/ERK pathway during mesodermal specification in frogs (Nutt et al., 2001; Sivak et al., 2005), similar inhibition of PLCγ might occur during WD elongation in chickens.

Recently, Attia et al. reported that FGF signals are crucial for WD elongation (Attia et al., 2015). However, they did not detect attractive activity by FGF8. The reason for the discrepancy between their results and ours is not known, although several possibilities are conceivable. The first relates to how the activities of FGF8 protein and drug inhibitors were validated: for the FGF8 transfection into cultured cells, we used the bidirectional promoter (pBI) (Watanabe et al., 2007), which drives *EGFP* and *FGF8* cDNAs simultaneously, allowing us to confirm high transfection efficiency by EGFP signal. The second possibility relates to how FGF8-producing cells and drug-soaked beads were implanted into embryos: in our experience, implants should be placed precisely in between the surface ectoderm and somatopleural mesoderm (as shown in Fig. 4F-H). If embedded too deep in the coelomic cavity or splanchnopleura, the implant would not influence WD elongation.

### Coordination between tissue morphogenesis and body growth

In this study, the FGF gradient has been shown to coordinate the leader and rear cells during WD elongation. This gradient is initiated by FGF8 produced in the tail bud, which is essential for the body elongation (Bénazéraf et al., 2010; Dubrulle and Pourquie, 2004). Thus, the body growth of the embryo coordinates the WD elongation, and this is mediated by FGF8.

In addition to the coordinated elongations between the body axis and WD, it is also known that the FGF8 gradient regulates the progression of somite segmentation (Dubrulle et al., 2001). Thus, the growth of the embryo body coordinates at least two types of morphogenesis – segmentation and WD elongation – in which FGF8 operates as a master coordinator. The coordination within this trinity can also explain the phenomenon at the molecular level that the WD leader is always located at sm –1 to –2, with the relative positions unchanged during body elongation. Importantly, our findings have revealed the coordination not only between multiple tissues, but also between different morphogenetic cells within the WD. It is remarkable that a macroscopic change, such as body axis elongation, is coordinately translated to morphogenetic changes at the microscopic level, such as the chemoattraction of leader cells and tubule formation of rear cells, both of which progress posteriorly with a regular interval between them. Of note, WD formation, in turn, appears to regulate Müllerian duct elongation, which also involves coordination between exploring front cells and epithelizing tubular cells (Orvis and Behringer, 2007). Along this line, an elegant study on mouse lung formation should be mentioned in which branching morphogenesis antagonizes alveolar differentiation, so that both events progress in a coordinated manner as the developing lung grows (Cunningham et al., 2013), and it awaits to be seen whether such morphogenetic coordination within the lung would also be controlled by body growth. From this viewpoint, it should be highlighted that diseases seen at the microscopic level (i.e. cellular dysfunction) might link to a macroscopic malformation (i.e. body growth), providing a new avenue for therapeutic treatments.

## MATERIALS AND METHODS

### Experimental animals

Fertilized chicken eggs were obtained from Shiroyama-Keien (Sagamihara, Japan). Embryos were staged according to Hamburger and Hamilton (1992) (HH) or somite number. All animal experiments were conducted with the ethical approval of Kyoto University (No. H2620).

### PKH labeling

The presumptive pronephric region of 10-somite embryos (10 sm) was labeled with PKH26 or PKH67 fluorescent cell linker (Sigma) as previously reported (Atsuta et al., 2013).

### Expression vectors

pCAGGS-GAP43-EGFP and pCAGGS-tTA were described previously (Atsuta et al., 2013). For pCAGGS-tdTomato, tdTomato was subcloned into the *Eco*RI-*Bgl*II sites of the pCAGGS plasmid (Niwa et al., 1991). For pCAGGS-GAP-EGFP-P2A, GAP43-EGFP and oligonucleotides encoding P2A (Kim et al., 2011) were subcloned into the *Mlu*I-*Xho*I and *Xho*I-*Eco*RI sites, respectively, of the pCAGGS plasmid. To obtain pCAGGS-GAP-EGFP-2A-H2B-mCherry, H2B-mCherry was ligated into the *Eco*RV site of pCAGGS-GAP-EGFP-2A. cDNAs for chicken *FGF8B* and *FGF10* were gifts of Drs H. Nakamura (Tohoku University) and H. Ohuchi (Okayama University), respectively. Each was subcloned into the *Mlu*I-*Nhe*I sites of pBI-TRE-EGFP (Watanabe et al., 2007) to obtain pBI-TRE-FGF8-EGFP and pBI-TRE-FGF10-EGFP. cDNA for chicken *GDNF* was isolated by PCR using primers (5′-3′): GDNF-F, ATAACGCGTATGTTACAAAGCATTTTTTC; GDNF-R, ATAGCTAGCTCAGACACATCCACACCAA. An amplified fragment was subcloned into the *Mlu*I-*Nhe*I sites of pBI-TRE-EGFP to obtain pBI-TRE-GDNF-EGFP.

### *In ovo* electroporation

*In ovo* DNA electroporation into WD cells was performed using CUY21 EX (BEX) as described (Atsuta et al., 2013).

### DNA transfection and preparation of DF1 cell aggregate

Chicken fibroblast-derived DF1 cells were maintained at 39°C with Dulbecco's modified Eagle's medium (Nissui) containing 10% fetal bovine serum (FBS/DMEM) and Penicillin-Streptomycin (Gibco). pBI-TRE-EGFP, pBI-TRE-FGF8-EGFP, pBI-TRE-FGF10-EGFP or pBI-TRE-GDNF-EGFP was co-transfected with pCAGGS-tTA into DF1 cells using Lipofectamine 2000 (Invitrogen) as previously described (Saito et al., 2012). Twenty-four hours after transfection, cells were transferred to a dish coated with 1% agarose to obtain cell aggregates (Tonegawa et al., 1997).

### Embryological manipulation

Surgical manipulations were performed with E2/HH13 embryos (about 20 sm). Transplantation of a donor tissue, cell aggregate or bead into a host embryo was performed using a tungsten needle. FGFs were delivered using heparin acrylic beads (Sigma) soaked overnight with 250 μg/ml human FGF4 (WAKO) or 250 μg/ml human FGF8B (WAKO) at 4°C. For chemical-soaked bead experiments, AG1X2 ion-exchange beads (formate form; BioRad) were incubated in 10 mM SU5402 (Calbiochem), 20 mM PD184352 (Sigma), 20 mM LY294002 (Sigma), 10 mM U73122 (Sigma), 20 mM SP600125 (Sigma) or 20 mM SB203580 (Sigma) for 2 h at room temperature before grafting.

### Immunohistochemistry

For immunological staining on histological sections, anti-laminin mouse monoclonal antibody (3H11; DSHB), anti-E-cadherin mouse monoclonal antibody (BD Biosciences, 610182) and anti-ZO-1 rabbit polyclonal antibody (Zymed, 40-2200) were used as described (Atsuta et al., 2013). Sections treated with antibodies were also exposed to DAPI (Sigma).

To detect diphosphorylated ERK (pERK), sections were treated with 3% H_2_O_2_ in TNT (0.1 M Tris-HCl, 0.15 M NaCl, 0.1% Tween 20, pH 7.5) for 30 min, and pre-blocked with 1% Blocking Reagent (Roche) in TNT for 1 h. The sections were incubated overnight at 4°C with 1:300 anti-pERK rabbit polyclonal antibody (Cell Signaling, 4370). They were then washed in TNT and incubated with 1:1000 anti-rabbit IgG horseradish peroxidase (HRP)-conjugated donkey antibody (GE HealthCare, NA9340-1ML) for 30 min. After washing in TNT, sections were reacted with the TSA Plus Cy3 system (PerkinElmer) for 5 min at room temperature. The reaction was terminated by washing in TNT, and sealed by FluorSave Reagent (Calbiochem) containing DAPI. Fluorescent images were obtained using an Axioplan 2 microscope with Apotome system (Carl Zeiss).

For whole-mount detection of pERK, embryos were fixed with 4% paraformaldehyde (PFA) in PBS overnight at 4°C. Fixed embryos were dehydrated with methanol and treated with 3% H_2_O_2_ in methanol for 4 h. After rehydration with PBS containing 0.1% Tween 20 (PBST), embryos were washed in PBS containing 0.5% Tween 20. After blocking with 5% FBS in PBST, they were incubated with 1:200 anti-pERK antibody for 48 h at 4°C. After washing in TBST (0.1 M Tris-HCl, 0.15 M NaCl, 0.5% Triton X-100, pH 7.5), they were incubated with 1:200 anti-rabbit IgG HRP-conjugated donkey antibody.

For DAB (3,3′-diaminobenzidine; WAKO) staining, specimens were washed with TBST and incubated with DAB/PBST for 20 min, then reacted with a 1:2000 dilution of 30% H_2_O_2_ for 5 min. For double staining of LIM1 protein and pERK, 1:200 anti-LIM1 mouse monoclonal antibody (4F2; DSHB) was used along with the anti-pERK antibody.

### Probes and *in situ* hybridization

Chicken cDNA fragments for *FGFR2* and *FGFR3* were isolated by PCR using primers (5′-3′): FGFR2-F, TTTACGCGTGAGAGCGTAGTCCCATCCGA; FGFR2-R, TGAGCTAGCTCATTTTGGATCCTCTGGCAGTTC; FGFR3-F, ATAGATATCATGTCTGAGGCGGGCGGCGG; FGFR3-R, ATAGATATCTCAGATGAAGAGGACTAAGCCAG. cDNAs of *LIM1*, *PEA3*, *FGF4* and *FGF8* were gifts of Drs H. Nakamura and K. Tamura (Tohoku University). cDNAs of *SEF* and *FGFR1* were isolated by Dr E. Shimokita (Nara Institute of Science and Technology, NAIST). Digoxigenin-labeled probes were prepared according to the manufacturer's instructions (Roche). Whole-mount *in situ* hybridizations were performed as previously described (Tonegawa et al., 1997).

### Whole-embryo culture and time-lapse imaging

Whole-mount embryo culture was performed as described (Atsuta et al., 2013). For cell tracking analyses, images were processed with the Manual Tracking Tool of ImageJ (NIH).

### *In vitro* migration assay

WD cells were dissected using tungsten needles and placed with FGF8-soaked beads or FGF8-expressing cells in a 35-mm glass-bottom dish (MATSUNAMI) coated with 10 ng/ml fibronectin (WAKO). The specimens were embedded in Matrigel (BD Biosciences) diluted with Opti-MEM (Invitrogen), and cultured for 6 h with FBS/DMEM.

### TUNEL assay

The TUNEL assay was performed with the In Situ Cell Death Detection Kit (Roche) according to the manufacturer's instructions.

## Supplementary Material

Supplementary Material
