# Supplementary material for: FGF8 coordinates tissue elongation and cell epithelialization during early kidney tubulogenesis
Source: Development. 2015 Jul 1;142(13):2329–37. doi: 10.1242/dev.122408 (PMC4510593; doi:10.1242/dev.122408)
Supplement: Supplementary Material [file supp_142_13_2329__index.html]

Supplementary Material 

# FGF8 coordinates tissue elongation and cell epithelialization during early kidney tubulogenesis

## DEV122408 Supplementary Material

- Supplementary Material
