# Supplementary material for: FGF8 coordinates tissue elongation and cell epithelialization during early kidney tubulogenesis
Source: Development. 2015 Jul 1;142(13):2329–37. doi: 10.1242/dev.122408 (PMC4510593; doi:10.1242/dev.122408)
Supplement: Supplementary Material [file supp_142.13.2329_DEV122408supp.pdf]

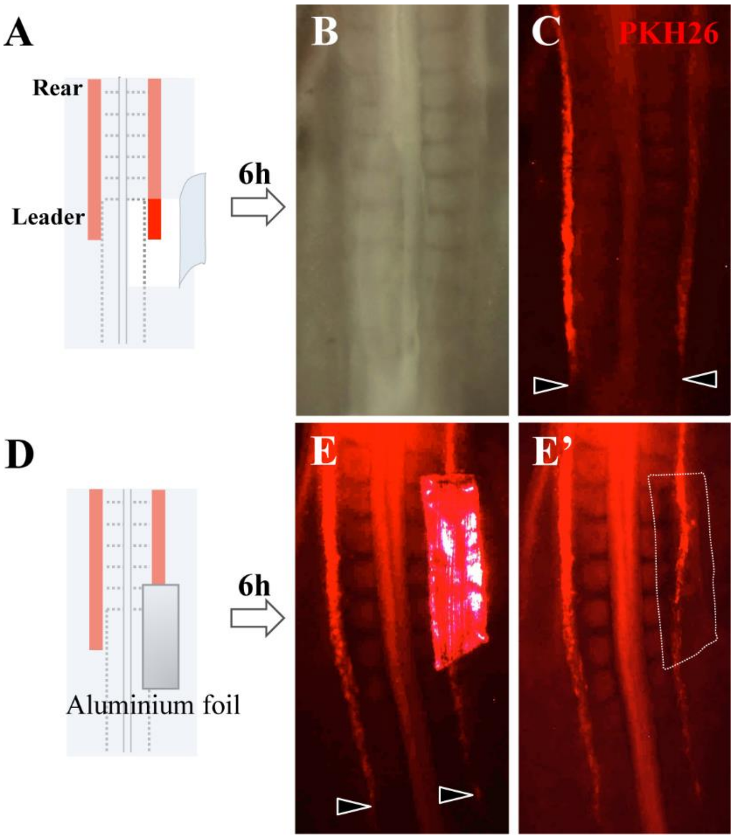

Figure S1. Signals from the surface ectoderm are dispensable for WD elongation. (A) Schematic diagrams showing that the surface ectoderm overlying leader cells was removed from an E2/HH13 embryo. (B, C) Caudal migration of WD on the treated side was comparable to the untreated side (black arrowheads). (D, E) A piece of aluminum foil was inserted in between the WD and surface ectoderm, resulting in no disturbed elongation of WD (black arrowheads).

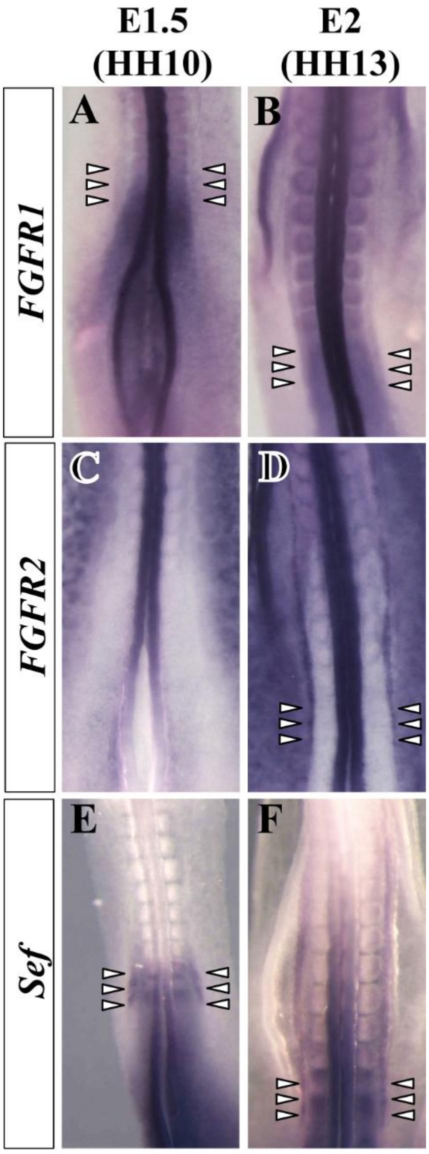

Figure S2. Expression patterns of *FGFR1*, *FGFR2* and *SEF*. Whole-mount in situ hybridization to show transcripts of *FGFR1* (A, B), *FGFR2* (C, D) and *SEF* (E, F) in E1.5/HH10 and E2/HH13 embryos. *FGFR1* and *SEF* were expressed in the WD at both stages, and *FGFR2* was expressed in E2/HH13 embryo (white arrowheads).

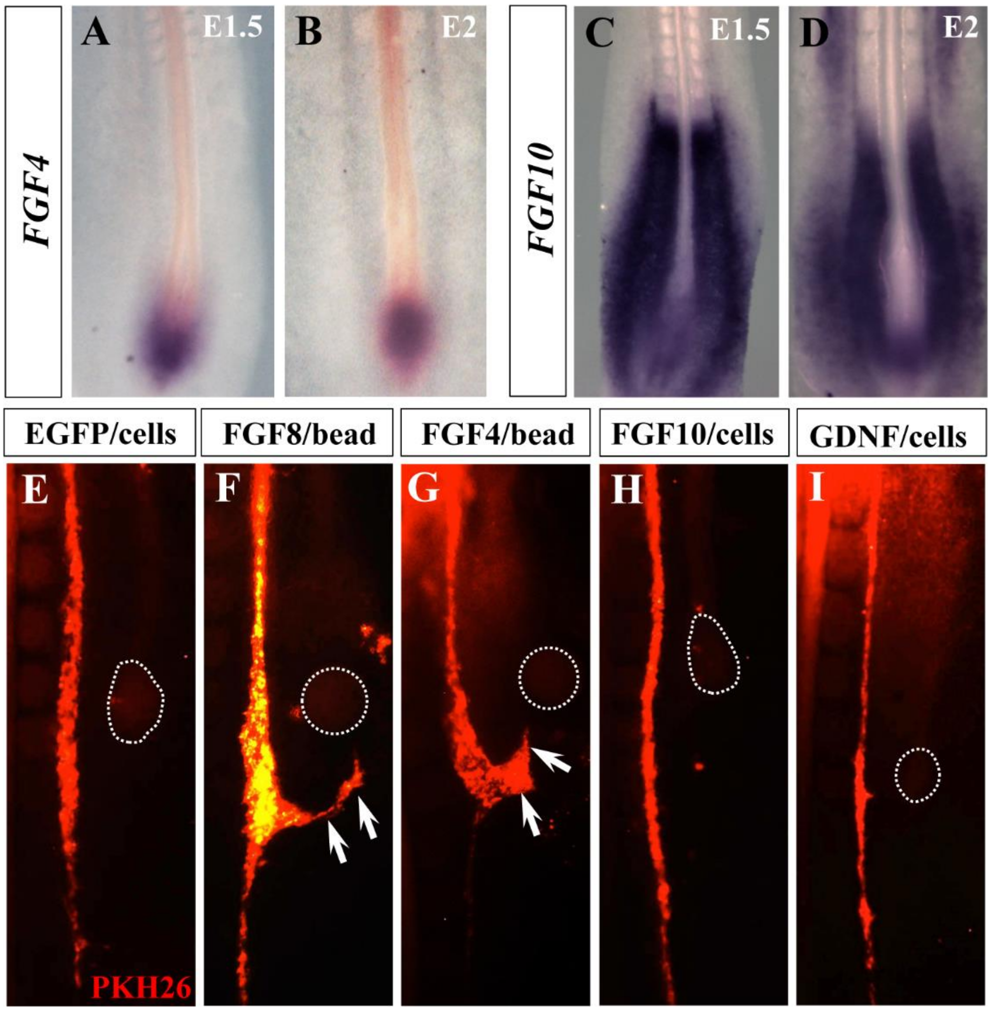

Figure S3. FGF8, FGF4, but not FGF10, attracted leader cells. Whole-mount in situ hybridization to show expression patterns of *FGF4* (A, B) and *FGF10* (C, D) in E1.5/HH10 and E2/HH13 embryos. An implanted FGF8-soaked or FGF4-soaked bead attracted leader cells ([F, G], arrows) whereas EGFP-expressing cells yielded no effect (E). (H, I) Neither FGF10-producing nor Glial-derived growth factor (GDNF)-producing DF1 cells showed attracting activity.

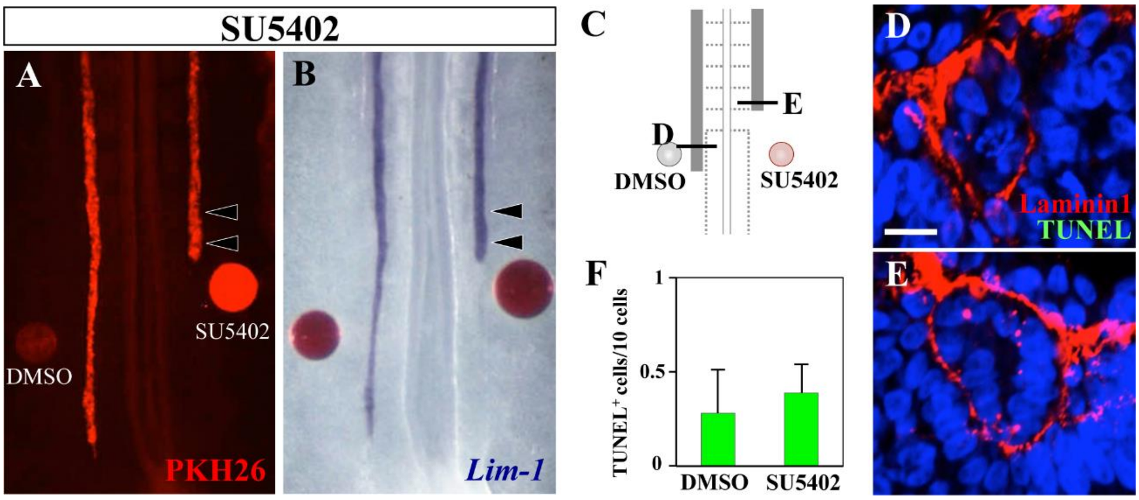

Figure S4. Inhibition of FGF signaling did not affect *LIM1* expression or cell survival. (A, B) Expression of *LIM1* mRNA was not downregulated by SU5402 treatment (arrowheads). (C-F) SU5402 did not excessively induce cell deaths. (D, E) Transverse sections indicated in (C) were double-stained with antibody for Laminin1 (red) and TUNEL (green). (F) Quantification of apoptotic cells within DMSO-treated (n = 63 cells in 8 embryos) or SU5402-treated leader cells (n = 121 cells in 8 embryos). Error bars represent SEM. Scale bars: 20 µm for (D).

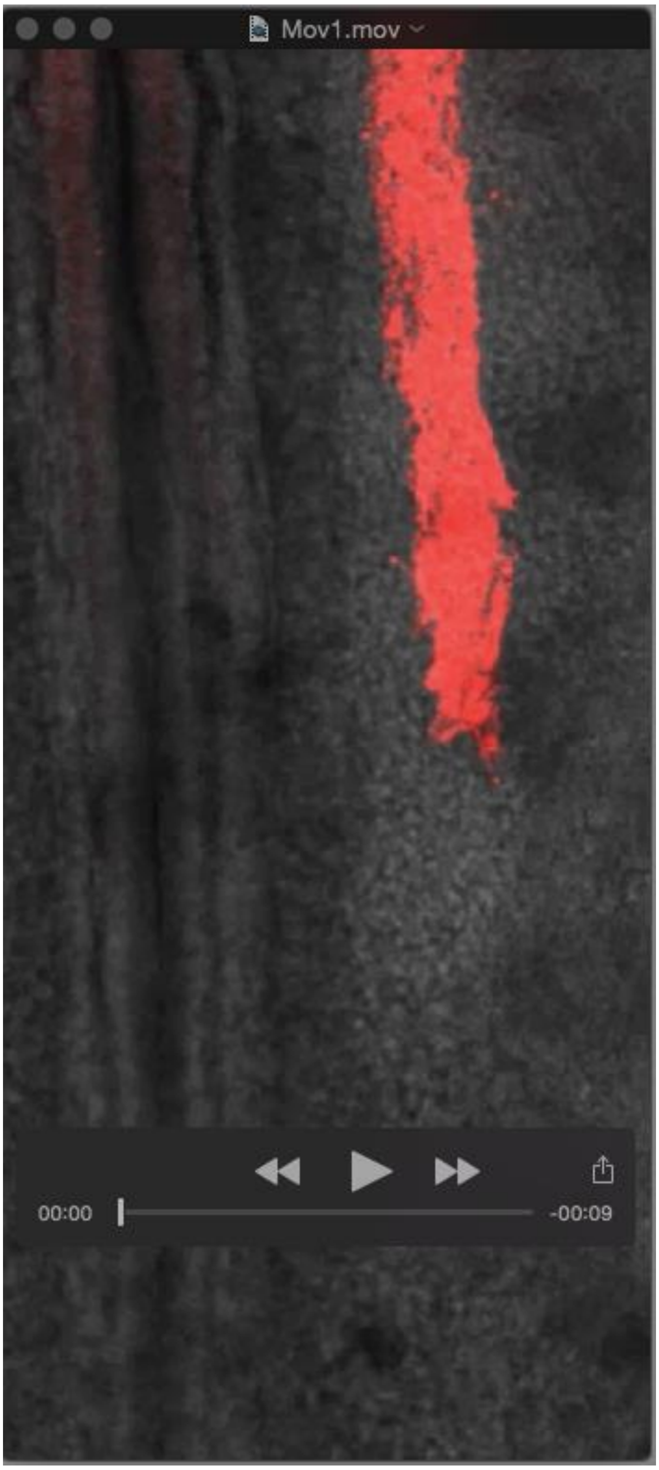

Movie 1. Time-lapse analyses using a cultured whole embryo, the WD of which was labeled with a red fluorescent dye PKH26. Frames were taken every 3 min with a 10 × Plan-Apochromat objective lens. Total movie length: 270 min.

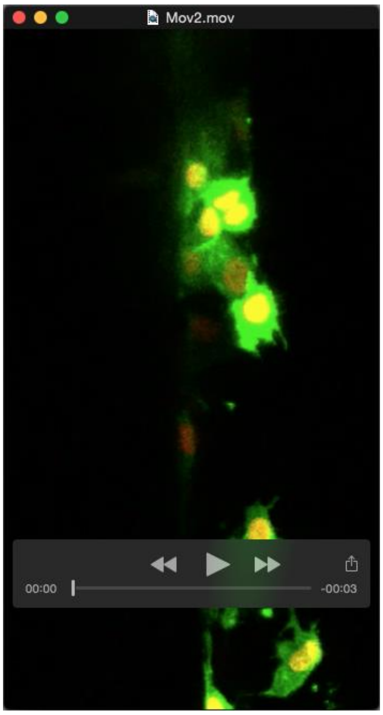

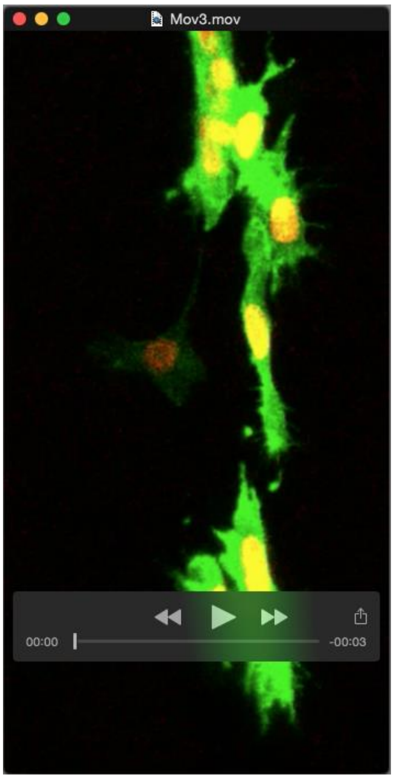

Movies 2, 3. In vivo time-lapse analyses for rear cells (Movie 2) and leader cells of WD (Movie 3), which were electroporated with pCAGGS-GAP-EGFP-P2A-H2B-mCherry. Frames were taken every 1 min with a 40× Plan-Apochromat objective lens. Total movie length: 30 min.

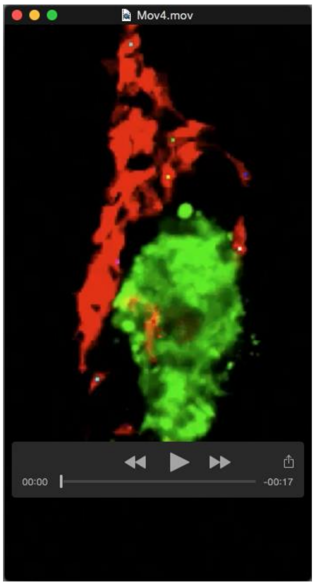

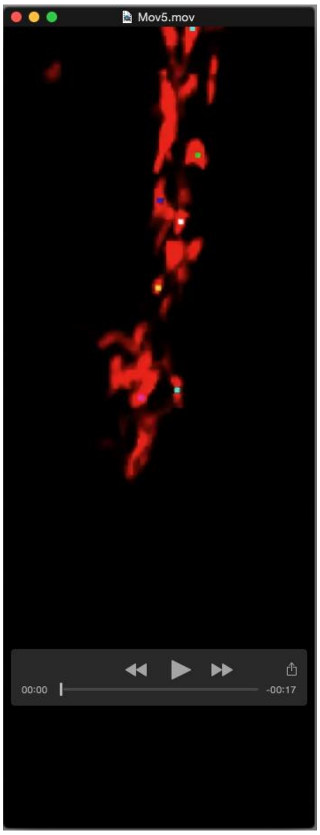

Movies 4, 5. Time-lapse analyses using cultured whole embryos where pCAGGS-tdTomato (red) was electroporated into WD. When FGF8-producing DF1 cells (green) were implanted, leader cells migrated toward the FGF8/DF1 (Movie 4). 10 cells in each movie were traced by digitally processed colors. Movie 5 is of a normal embryo. Frames were taken every 3 min with a 10× Plan-Apochromat objective lens. Total movie length: 180 min.

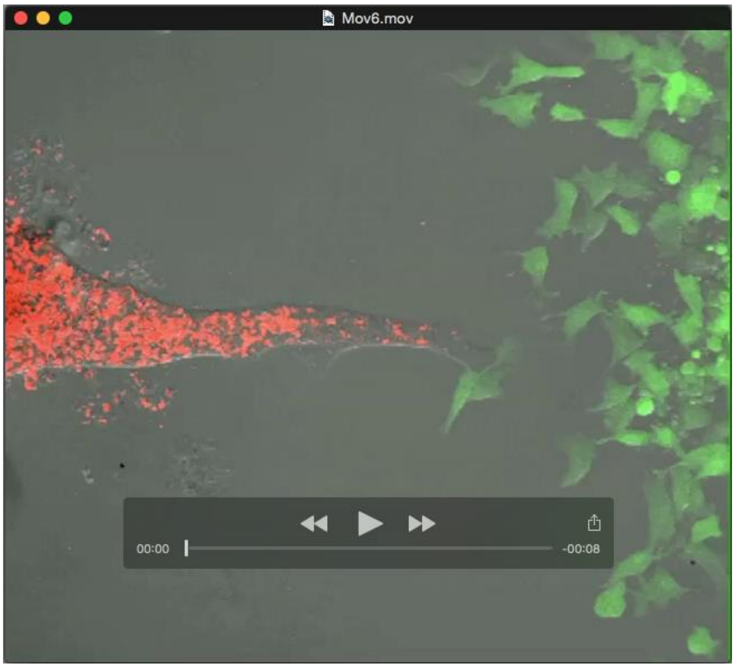

Movie 6. *In vitro* live-imaging and chemoattraction assay using leader cells (red) taken from PKH26-labeled WD co-cultured with FGF8-EGFP-producing DF1 cells (green). Frames were taken every 5 min with a 20× Plan-Apochromat objective lens. The length of each movie: 300 min.

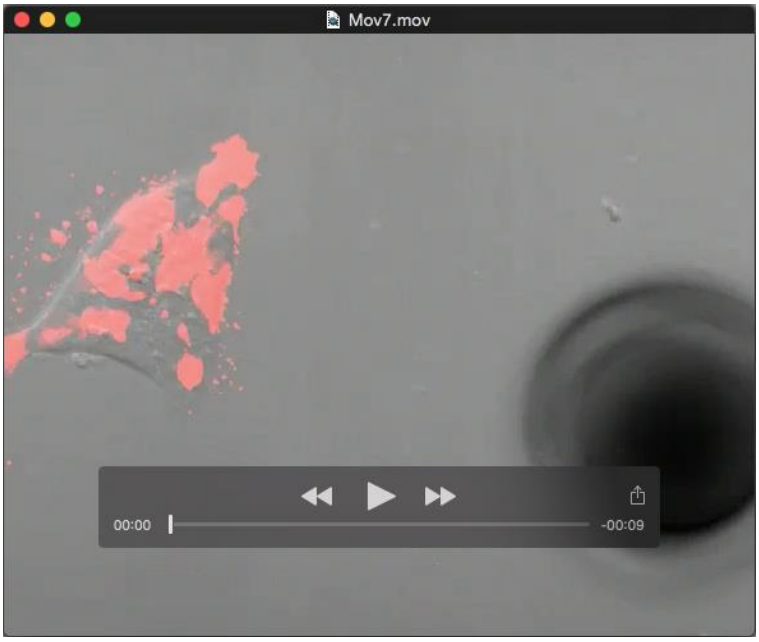
Mov7.mov
00:00
-00:09

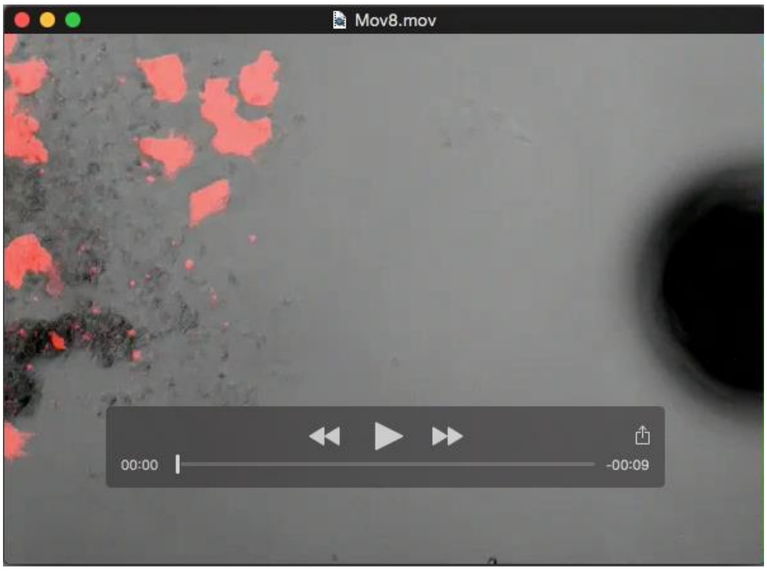
Mov8.mov
00:00
-00:09

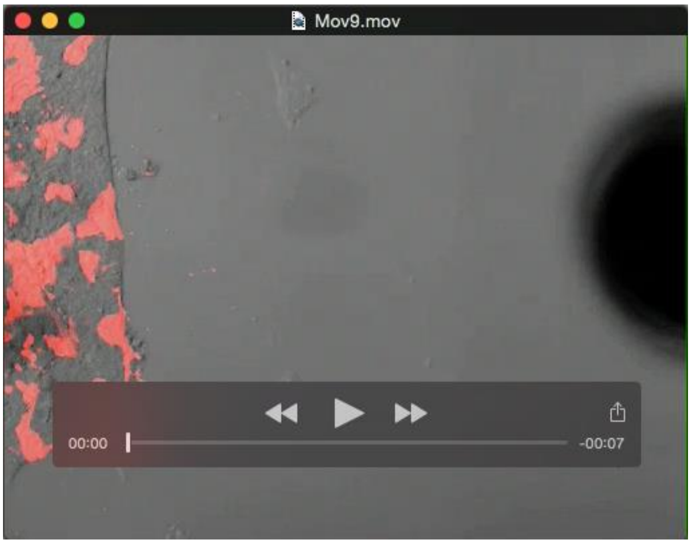

Movie 7-9. *In vitro* chemoattraction assay using leader cells co-cultured in Matrigel with an FGF8-soaked bead (Movie 7), DMSO-soaked bead (Movie 8) and FGF8-soaked bead in the presence of 2μM SU5402 (Movie 9). pCAGGS-tdTomato (red) was electroporated into WD to visualize individual cells prior to the culture. Frames were taken every 5 min with a 20× Plan-Apochromat objective lens. The length of each movie: 360 min.

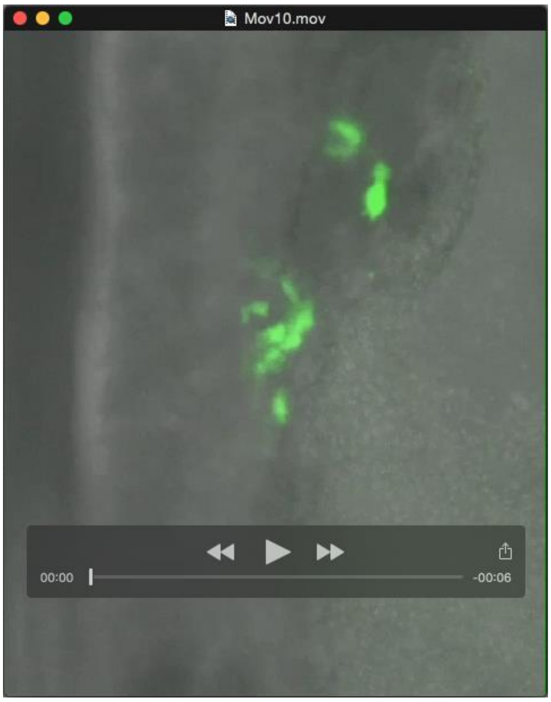

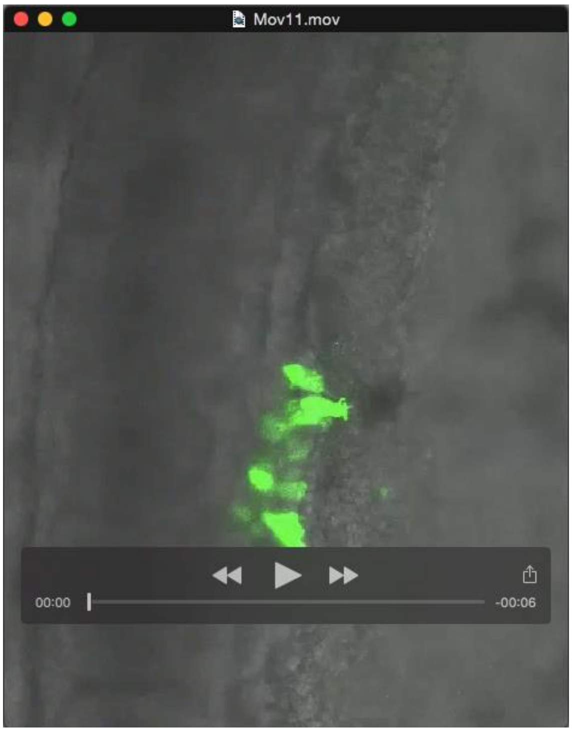

Movies 10, 11. Time-lapse analyses showing that forced-epithelialized cells by SU5402 could be transformed into motile cells when transplanted into the leader region (Movie 10), whereas cells transplanted into the rear region remained static (Movie 11). Frames were taken every 1 min with a 20× Plan-Apochromat objective lens. The length of each movie: 60 min.
